# Safety of tenecteplase versus alteplase for intravenous thrombolysis in acute ischemic stroke patients with direct oral anticoagulation: experience from a German stroke center

**DOI:** 10.1186/s42466-025-00450-8

**Published:** 2025-11-14

**Authors:** Lena Mers, Anna Bogdanova, Alexander Sekita, Ludwig Singer, Manuel Schmidt, Bernd Kallmuenzer, Stefan Schwab, Stefan T. Gerner

**Affiliations:** 1 Department of Neurology, University Hospital Erlangen, Schwabachanlage 6, 91054 Erlangen, Germany; 2 Department of Neuroradiology, University Hospital Erlangen, Schwabachanlage 6, 91054 Erlangen, Germany

**Keywords:** Ischemic stroke, Intravenous thrombolysis, Tenecteplase, Alteplase, Direct oral anticoagulation, Safety, Intracranial hemorrhage

## Abstract

**Background:**

Despite current guidelines recommending against intravenous thrombolysis (IVT) in acute ischemic stroke (AIS) patients with direct oral anticoagulants (DOAC) within prior 48 h, latest real-world data indicate no increased bleeding risk. However, these observations are based mainly on alteplase (rt-PA), whereas data for tenecteplase (TNK) are scarce.

**Methods:**

We retrospectively compared data from our stroke registry of AIS-patients with DOAC (intake within the last 48 h), who received IVT either with rt-PA or TNK without prior antagonization. The primary outcome was the rate of symptomatic intracranial hemorrhage (sICH) per SITS-Most criteria. Secondary outcomes included the rate of any ICH or major bleeding, rate of mortality, neurological and functional outcome at discharge.

**Results:**

82 AIS-patients were included, with 42 patients receiving TNK und 40 patients receiving rt-PA. Median age was 83 y for TNK patients and 82 y for rt-PA patients. Median NIHSS score at admission for TNK was 9 points for both groups (*p* = 0.61). Median drug-specific DOAC plasma level was 49 ng/mL for TNK *versus* 24 ng/mL for rt-PA (*p* = 0.04). We found no statistically significant increased risk for neither sICH (TNK 2.4% vs. rt-PA 2.5%; *p* = 1), nor for other safety outcomes for TNK-treated patients compared with rt-PA. The rate of excellent functional outcome (TNK 61.9% vs. rt-PA 52.5%) was similar among both groups. High drug-specific DOAC plasma levels were not related to an increased rate of hemorrhagic complications in our cohort.

**Conclusion:**

We report no increased rate of (s)ICH for TNK based IVT compared with rt-PA in AIS-patients with DOAC, indicating a similar safety profile. Moderate to high drug-specific DOAC levels were no surrogates for hemorrhagic complications, supporting the implementation of specific Standard Operating Procedures for IVT in DOAC-treated patients. Contrary to previous studies, we did not observe an increased rate of early recanalization of LVO in TNK-treated patients in this small single-center cohort.

**Trial registration:**

n/A.

**Supplementary Information:**

The online version contains supplementary material available at 10.1186/s42466-025-00450-8.

## Introduction

In the treatment of acute ischemic stroke (AIS) intravenous thrombolysis (IVT) is essential in evidence based standard of acute recanalization therapy [1, 2], improving clinical outcomes [3–5]. As alteplase (rt-PA) has been the global standard, tenecteplase (TNK) has recently been established as an alternative thrombolytic agent [6] with a similar safety profile [7–15], but presumed distinct pharmacological and practical advantages over rt-PA [16–18].

Within the patient population eligible for IVT, at least one in six patients is being treated with direct oral anticoagulants (DOAC) [19]. Up to 20% of AIS-patients were on DOAC prior to the index event [20], and number of prescriptions is continuously increasing [4]. However, most international guidelines do not recommend IVT in patients with DOAC-intake within 48 h prior to AIS [21, 22], primarily due to safety concerns such an increased rate of symptomatic intracranial hemorrhage (sICH) [23]. To date, oral anticoagulation is therefore considered as the most common absolute contraindication for IVT [24].

In contrast, latest real-world data indicate no increased risk of IVT-associated hemorrhagic complications [2, 25–29]. For an overview of literature see [30]. However, these data are based mainly on rt-PA, whereas data for TNK are scarce [19]. As TNK is increasingly being adopted in stroke centers worldwide, it is important to assess the safety and efficacy of TNK in this large patient population.

The main objective of our study was to analyze safety and efficacy of TNK-based IVT in AIS-patients with DOAC receiving TNK (optionally endovascular thrombectomy [EVT]) in the absence of prior antagonization compared with rt-PA. The primary outcome was the rate of sICH, secondary outcomes were rates of any intracranial or other major bleeding and in-hospital mortality, as well as neurological and functional outcome at discharge.

## Methods

### Study design, patient selection and data acquisition

This retrospective, single-center observational study included consecutive patients with acute ischemic stroke (AIS) who received intravenous thrombolysis (IVT) with either tenecteplase (TNK) or alteplase (rt-PA) at the University Hospital Erlangen between March 2023 and June 2025. Eligible patients had confirmed AIS and reported intake of a direct oral anticoagulant (DOAC) within 48 h prior to admission. Patients were assigned to treatment groups according to the institutional time-based switch from rt-PA (until March 2024) to TNK (from April 2024). All data were retrieved from the institutional stroke registry and patient charts, as described previously [31].

### Baseline characteristics

For baseline characteristics, demographic data, vascular risk factors, cardiovascular comorbidities, and premorbid functional status (mRS) were collected. Stroke-related parameters included neurological severity on admission (NIHSS), onset-to-door and onset-to-needle times, categorized as ≤ 4.5 h versus > 4.5 h or unknown. The presence and location of large vessel occlusion (internal carotid, M1/M2 MCA, posterior cerebral, vertebral, or basilar artery) were recorded. DOAC-related variables comprised the specific agent, indication, dosage, and plasma concentration on admission, measured by drug-specific anti-Xa (STA^®^ - Liquid Anti-Xa, anti-Xa chromogenic assay) or ecarin chromogenic assays (STA^®^-ECA II), and referred to as the drug-specific DOAC level throughout the manuscript.

### Local stroke management

Until 05/2024 rt-PA was used as the standard thrombolytic agent, in 06/2024 TNK was implemented into routine practice at our stroke center. IVT was performed according to current guidelines using rt-PA (0.9 mg/kg; max 90 mg) or TNK (0.25 mg/kg; max 25 mg). Non-contrast or multimodal CT imaging guided treatment decisions. According to our standard operating procedure, IVT was recommended when the drug-specific DOAC plasma concentration was ≤ 100 ng/mL; at higher levels, treatment decisions were individualized based on clinical judgment and imaging findings.

### Imaging and outcome assessment

Baseline imaging (Siemens SOMATOM x.ceed) included non-contrast CT and, when indicated, CT-angiography/perfusion. Follow-up scans were obtained 24 h after IVT or upon neurological deterioration. sICH was defined according to SITS-MOST criteria (NIHSS ≥ 4 plus parenchymal hematoma type 2 [32]). Secondary endpoints comprised any ICH, major bleeding (ISTH definition [33]), in-hospital mortality, early neurological improvement (NIHSS reduction ≥ 4 or NIHSS = 0 within 72 h), and functional outcome at discharge (mRS 0–1 = excellent, 0–2 = favorable).

### Statistical analysis

Analyses were performed in SPSS v28 (IBM). Categorical variables were compared using χ² or Fisher’s exact tests; continuous variables using the Mann–Whitney U test. Two-sided *p* < 0.05 was considered statistically significant. Subgroup analyses examined associations of outcomes with (i) IVT time window (≤ 4.5 h vs. > 4.5 h/unknown), (ii) drug-specific DOAC level (≤ 50 vs. > 50 ng/mL), and (iii) EVT (yes/no). As a sensitivity analysis, we performed propensity-score matching (nearest-neighbor, caliper 0.2) on age, heart failure, diabetes, and DOAC level to address baseline imbalances. No multiple-imputation was used. Given the rarity of sICH and the actual group sizes (TNK *n* = 42; rt-PA *n* = 40), the study is underpowered to detect small differences; with an observed sICH rate ≈ 2.5%, only large absolute differences ( ≈ ≥ 20% points) would be detectable at 80% power (α = 0.05). Accordingly, non-significant findings should be interpreted with caution and viewed as exploratory.

## Results

**Fig. 1:**
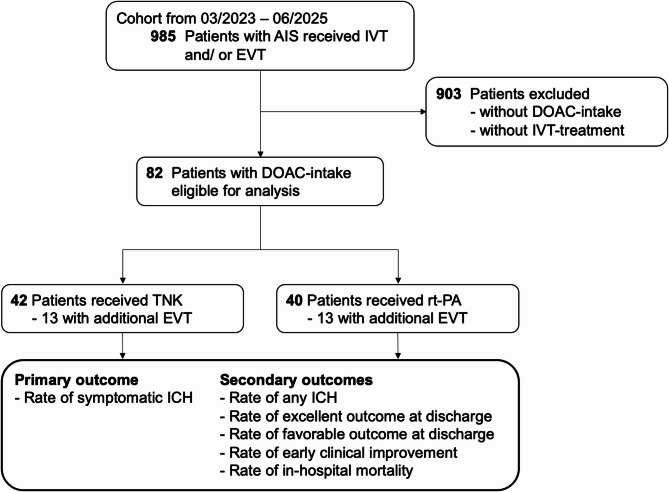
Flow chart of the study participants. Overall, data from 985 patients with AIS - admitted between 03/2023 and 06/2025 - who received recanalization therapy with IVT and/or EVT were identified. After exclusion of all patients with neither reported DOAC-intake nor IVT-treatment, 82 patients remained for analyses of primary and secondary outcomes and were dichotomized according to fibrinolytic agent (TNK *n* = 42; rt-PA *n* = 40). Abbreviations: AIS indicates acute ischemic stroke; DOAC, direct oral anticoagulation; EVT, endovascular treatment; ICH, intracerebral hemorrhage; IVT, intravenous thrombolysis; rt-PA, alteplase; TNK, tenecteplase

The study flow chart (Fig. 1) outlines patient inclusion. Among 985 patients with suspected AIS treated with IVT and/or EVT over 28 months, we excluded those without IVT or without prior DOAC-intake. 82 patients met inclusion criteria: 40 received rt-PA and 42 TNK; 13 in each group also underwent EVT.

### Demographic characteristics

Baseline characteristics are summarized in Table 1. The median age was 83 y within the TNK-group and 82 y in the rt-PA-group. Females represented 56.1% of the cohort. Premorbid mRS score distribution was balanced with a median of 2 for TNK and rt-PA (IQR = 0–3; *p* = 0.6). Both groups did mostly not differ in the rate of cardiovascular comorbidities, except a higher prevalence of heart failure and diabetes mellitus type II among TNK-treated patients (see Table 1).

**Table 1:** Baseline characteristics and stroke features

Parameter	AIS-patients on DOAC treated with IVT			*p* value
	Overall *n* = 82	TNK *n* = 42 (51.2%)	rt-PA *n* = 40 (48.8%)	
Age, y; median (IQR)	82 (74–88)	83 (70–86)	82 (77–89)	0.35
- Patients aged > 80 years; n (%)	45 (54.9%)	24 (57.1%)	21 (52.5%)	0.88
Female sex; n (%)	46 (56.1%)	24 (57.1%)	22 (55.0%)	1
**Prior medical history**				
Premorbid mRS Score; median (IQR) - Premorbid mRS score 0–1, n (%)	2 (0–3) 34 (41,5%)	2 (0–3) 17 (40.5%)	2 (0–3) 17 (42.5%)	0.57 1
- Premorbid mRS score 0–1, n (%)	34 (41,5%)	17 (40.5%)	17 (42.5%)	1
Previous Stroke or TIA; n (%)	24 (29.3%)	13 (31.0%)	11 (27.5%)	0.81
Coronary heart disease; n (%)	19 (23.2%)	9 (21.4%)	10 (25.0%)	0.8
Heart failure; n (%)	14 (17.1%)	12 (28.6%)	2 (5.0%)	0.007
Hypertension; n (%)	76 (92.7%)	39 (92.9%)	37 (92.5%)	1
Diabetes mellitus Type II; n (%)	28 (34.1%)	19 (45.2%)	9 (22.5%)	0.04
Vascular disease*; n (%)	17 (20.7%)	9 (21.4%)	8 (20.0%)	1
Atrial fibrillation; n (%)	76 (92.7%)	39 (92.9%)	37 (92.5%)	1
CHA2DS2-VASc-Score; median (IQR)	4 (4–6)	5 (3–6)	4 (4–6)	0.69
**DOAC characteristics**				
Agent; n (%)				
- Apixaban	52 (63.4%)	26 (61.9%)	26 (65.0%)	0.82
- Edoxaban	20 (24.4%)	11 (26.2%)	9 (22.5%)	0.8
- Rivaroxaban	8 (9.8%)	5 (11.9%)	3 (7.5%)	0.71
- Dabigatran	2 (2.4%)	0	2 (5.0%)	0.24
Drug specific DOAC level, ng/mL; median (IQR)	35 (22–72)	49 (24–82)	24 (22–65)	0.04
- < 30 ng/mL; n (%)	36 (44.4%)	15 (35.7%)	21 (53.8%)	0.12
- 30–50 ng/mL; n (%)	13 (16.0%)	7 (16.7%)	6 (15.4%)	1
- 51–100 ng/mL; n (%)	25 (30.9%)	16 (38.1%)	9 (23.1%)	0.16
- > 100 ng/mL; n (%)	7 (8.6%)	4 (9.5%)	3 (7.7%)	1
**Laboratory data; median (IQR)**				
INR	1.13 (1.06–1.24)	1.15 (1.06–1.24)	1.09 (1.06–1.24)	0.31
pTT, s	33 (31–35)	33 (31–36)	33 (31–35)	0.87
**Stroke characteristics**				
NIHSS on admission; median (IQR)	9 (4–14)	9 (4–13)	9 (4–15)	0.61
Stroke severity; n (%)				
- Mild (NIHSS 1–4)	26 (31.7%)	13 (31.0%)	12 (32.5%)	
- Moderate (NIHSS 5–15)	40 (48.8%)	22 (52.4%)	18 (45.0%)	
- Severe (NIHSS > 15)	16 (19.5%)	7 (16.7%)	9 (22.5%)	
Symptom-onset-to-door, min; median (IQR)	137 (64–293)	94 (58–288)	163 (72–416)	0.26
- Early time window (≤ 4.5 h); n (%)	56/79 (70.9%)	28/40 (70.0%)	28/39 (71.8%)	1
- Late time window (> 4.5 h) or unknown; n (%)	23/79 (29.1%)	12/40 (30.0%)	11/39 (28.2%)	
Symptom-onset-to needle, min; median (IQR)	196 (132–358)	160 (120–357)	215 (140–480)	0.26
- Early time window (≤ 4.5 h); n (%)	50 (61.0%)	28 (66.7%)	22 (55.0%)	0.37
- Late time window (> 4.5 h); n (%)	32 (39.0%)	14 (33.3%)	18 (45.0%)	
Dose of thrombolytic agent, mg; median (IQR)		20 (17.5–25)	63 (54–75)	
Large vessel occlusion**; n (%)	35 (42.7%)	15 (35.7%)	20 (50.0%)	0.26
- Anterior circulation	31 (37.8%)	15 (35.7%)	16 (40.0%)	0.08
- Posterior circulation	4 (4.9%)	0	4 (10.0%)	
EVT treatment; n (%)	26 (31.7%)	13 (31.0%)	13 (32.5%)	1
- Onset to groin, min; median (IQR)	253 (126–442)	174 (126–292)	274 (114–508)	0.76

*defined as the presence of coronary artery disease, peripheral artery disease or aortic plaque

** Internal Carotid Artery, M1/M2 segment of the Middle Cerebral Artery, Posterior Cerebral Artery, Vertebral Artery, Basilar Artery

Abbreviations: AIS indicates acute ischemic stroke; DOAC, direct oral anticoagulant; EVT, endovascular treatment; IQR, interquartile range; IVT, intravenous thrombolysis; mRS, modified Rankin Scale; NIHSS, National Institutes of Health Stroke Scale; TIA, transient ischemic attack; rt-PA, alteplase; TNK, tenecteplase

### DOAC characteristics

Most patients (*n* = 52) were treated with apixaban, followed by edoxaban (*n* = 20), rivaroxaban (*n* = 8), and dabigatran (*n* = 2). The median drug-specific DOAC level at admission was significantly higher for the TNK-group, with 49 ng/mL (IQR = 24–82 ng/mL) for TNK and 24 ng/mL (IQR = 22–65) for rt-PA (*p* = 0.04). DOAC characteristics are shown in Table 1.

### Stroke characteristics

Patients from both groups presented with a NIHSS score of 9 points (TNK IQR = 4–13; rt-PA IQR = 4–15; *p* = 0.61). 70.0% of TNK-patients and 71.8% of rt-PA-patients presented in the ≤ 4.5 h time window (*p* = 1). LVO - most frequent in the M2 segment of the middle cerebral artery - was present in 35.7% % of TNK-treated patients and in 50.0% cases of the rt-PA-group (*p* = 0.26).

### In-hospital outcomes

#### Hemorrhagic complications

Hemorrhagic complications are summarized in Table 2 and supplemental Fig. 1a. Overall, ICH occurred in 6 patients (7.3%), with 4 patients from the TNK-group and 2 patients who received rt-PA (TNK 9.5%; rt-PA 5.0%; *p* = 0.68). PH2 was rare, with one case each for TNK and rt-PA (TNK 2.4%; rt-PA 2.5%; *p* = 1). sICH according to SITS-Most criteria was observed in one patient per group (TNK 2.4%; rt-PA 2.5%; *p* = 1). Subanalyses demonstrated no association between additional EVT and hemorrhagic complications (supplemental Table 1). Moreover, neither higher DOAC plasma concentrations > 50 ng/mL, nor extended time window indicated an increased bleeding risk (supplemental Table 1).

**Table 2:** Radiological and clinical outcomes

Parameter	AIS-patients on DOAC treated with IVT			*p* value
	Overall *n* = 82	TNK *n* = 42 (51.2%)	rt-PA *n* = 40 (48.8%)	
**Hemorrhagic complications;** ***n*** ** (%)**				
Any intracerebral hemorrhage	6 (7.3%)	4 (9.5%)	2 (5.0%)	0.68
Heidelberg Bleeding Classification				
- 1a	2 (2.4%)	2 (4.8%)	0	0.74
- 1c	1 (1.2%)	0	1 (2.5%)	
- 2	2 (2.4%)	1 (2.4%)	1 (2.5%)	
- 3	1 (1.2%)	1 (2.4%)	0	
- PH2	2 (2.4%)	1 (2.4%)	1 (2.5%)	1
Symptomatic intracerebral hemorrhage (SITS-MOST criteria	2 (2.4%)	1 (2.4%)	1 (2.5%)	1
Major bleeding (ISTH criteria)	1 (1.2%)	1 (2.4%)	0	1
**Clinical outcomes; n (%)**				
Early major NIHSS improvement (≥ 4 points within 72 h or NIHSS = 0)	35 (43.8%)	17 (40.5%)	19 (47.5%)	0.66
- Within 24 h**	25/35 (71.4%)	12 (70.6%)	14/18 (73.7%)	1
Early major NIHSS deterioration* (≥ 4 points within 72 h)	9/81 (11.1%)	5 (11.9%)	4/39 (10.3%)	1
- Within 24 h**	8/9 (88.9%)	5/5 (100%)	3/4 (75.0%)	0.44
**Radiological outcomes; n (%)**				
Infarct demarcation on follow-up imaging	32/81 (39.5%)	15 (35.7%)	17/39 (43.6%)	0.5
EVT treatment				
- Early complete vessel recanalization before EVT	0	0	0	1
- mTICI 2b-3**	24/26 (92.3%)	12/13 (92.3%)	12/13 (92.3%)	1

*except for intubated patients due to EVT

**relative frequency distribution refers to the respective subgroup

Abbreviations: AIS indicates acute ischemic stroke; DOAC, direct oral anticoagulant; EVT, endovascular treatment; ISTH, International Society on Thrombosis and Haemostasis; IVT, intravenous thrombolysis; mTICI, modified thrombolysis in cerebral infarction; NIHSS, National Institutes of Health Stroke Scale; PH, parenchymatous hematoma; rt-PA, alteplase; TNK, tenecteplase

#### Clinical and neuroradiological outcomes

Further in-hospital outcomes comparing TNK- and rt-PA treated patients are shown in Table 2 and supplemental Fig. 1b. Neurological improvement within 72 h after admission was observed in 17 patients with TNK-based IVT compared with 19 patients treated with rt-PA (TNK 40.5%; rt-PA 47.5%; *p* = 0.66). Neurological improvement was more likely to occur in case of ≤ 4.5 h time window and a low drug-specific DOAC level, being independent from recanalization treatment (see supplemental Table 1). The Propensity Score Matching analysis (TNK *n* = 30; rt-PA *n* = 30) showed rates of hemorrhagic complications and clinical outcomes being consistent with our primary analyses (see supplemental Table 2).

### Clinical outcomes at discharge

Outcomes at discharge are summarized in Table 3 and supplemental Fig. 2. The median mRS score for both groups was 3 (TNK IQR = 1–4; rt-PA IQR 2–4; *p* = 0.93). 26 TNK-patients achieved an excellent functional outcome (mRS score 0–1 or premorbid mRS score) compared with 21 rt-PA-patients (TNK 61.9%; rt-PA 52.5%; *p* = 0.5). In-hospital mortality rate was 14.3% for TNK and 12.5% for rt-PA (TNK *n* = 6; rt-PA *n* = 5; *p* = 1; Table 3). One patient’s death from each group was a consequence of sICH. Three patients died due to respiratory insufficiency and cardiovascular failure, two of whom due to Non-ST-Elevation Myocardial Infarction. Five patients died after a palliative procedure was initiated either due to concurrent complications (*n* = 3; i.e., pneumonia, acute renal failure) or space-occupying stroke (*n* = 2).

**Table 3:** Outcomes at hospital discharge

Parameter	AIS-patients on DOAC treated with IVT			*p* value
	Overall *n* = 82	TNK *n* = 42 (51.2%)	rt-PA *n* = 40 (48.8%)	
mRS score; median (IQR)	3 (2–4)	3 (1–4)	3 (2–4)	0.93
- Excellent neurological recovery (mRS score 0–1 or premorbid mRS score); n (%)	47 (57.3%)	26 (61.9%)	21 (52.5%)	0.5
- Favorable neurological recovery (mRS score 0–2 or back to premorbid mRS score); n (%)	50 (61.0%)	26 (61.9%)	24 (60.0%)	1
Mortality; n (%)	11 (13.4%)	6 (14.3%)	5 (12.5%)	1
**Discharge destination; n (%)**				
Rehabilitation	23 (28.0%)	15 (35.7%)	8 (20.0%)	0.12
Nursing home, Home care	8 (9.8%)	3 (7.1%)	5 (12.5%)	
Home	34 (41.5%)	13 (31.0%)	21 (52.5%)	
Other clinic	6 (7.3%)	5 (11.9%)	1 (2.5%)	

Abbreviations: AIS indicates acute ischemic stroke; DOAC, direct oral anticoagulant; IQR, interquartile range; IVT, intravenous thrombolysis; mRS, modified Rankin Scale; rt-PA, alteplase; TNK, tenecteplase

## Discussion

To our knowledge, this is the first study systematically comparing TNK- and rt-PA-based intravenous thrombolysis in acute ischemic stroke (AIS) patients with recent DOAC intake. We did not observe an increased risk of (s)ICH, other hemorrhagic complications or mortality of TNK-treated patients compared to rt-PA, noting an overall low complication rate for both thrombolytics.

### Safety of IVT in AIS-patients with DOAC

Depending on the definition, approximately 2 to 7% of patients undergoing IVT develop sICH, with fatal outcomes in 1.5 to 2% [34]. In our cohort, the observed sICH rate (~ 2.5%) was comparable between TNK and rt-PA and consistent with recent studies reporting similar or lower rates among DOAC-treated patients without prior antagonization [2, 23, 25, 35, 36, 37, 38].

We found no association between higher drug-specific DOAC plasma levels and sICH, even at concentrations >100 ng/mL. Although the small sample size limits precision, this observation aligns with previous multicenter reports [2, 23, 28]. Kleeberg et al. [28] analyzed 35 DOAC-treated patients receiving IVT (with or without EVT). The median DOAC level was 24 ng/mL; 89% of dabigatran cases received idarucizumab. ICH occurred in 20%, including 3% sICH, with several hemorrhages observed despite low DOAC levels (< 30 ng/mL). In Meinel et al. [2], who assessed drug-specific DOAC levels in 225 patients, a median of 21 ng/mL for patients treated with direct factor Xa-inhibitor, and 83 ng/mL for those treated with dabigatran were found. The authors observed no risk of sICH after IVT. Notably, median DOAC levels in our cohort were higher than in these studies, yet without excess bleeding.

Overall, data on pre-IVT coagulation assays remain limited, and cutoff thresholds for “safe” DOAC levels vary across institutions [19, 39]. Our findings support the pragmatic use of drug-specific DOAC level assessment to identify eligible patients, given that non-adherence and subtherapeutic dosing are frequent in this population [40].

### Safety and efficacy of TNK in comparison to rt-PA

Regarding safety, we found comparable rates of clinically relevant hemorrhagic complications for TNK and rt-PA. This aligns with current evidence showing similar sICH and mortality rates for both agents in non-anticoagulated patients [7–17, 41]. Existing data on IVT in DOAC-treated patients are largely rt-PA–based, reporting sICH rates between 1 and 4% [2, 25, 26, 34]. In comparison, our cohort showed a similar sICH risk despite higher age, comorbidity burden, and premorbid disability, indicating a reassuring safety profile also for TNK.

Evidence on TNK use in anticoagulated stroke remains scarce. Trials in non-anticoagulated populations report sICH rates of 2–5% [11, 12, 15, 41], while only one large analysis by Meinel et al. [2] included 51 TNK-treated DOAC patients—most after idarucizumab reversal—without specific subgroup results. Our data therefore add unique real-world evidence suggesting no excess hemorrhagic risk with TNK in DOAC-treated AIS. Ongoing multicenter trials (PASSION [EUCT 2025-521762-99], DO-IT [NCT06571149; registered 22/08/2024]) will further define its safety in this population. Further, we found no signal for higher early recanalization with TNK, possibly because elevated DOAC levels might attenuate stroke severity and obscure potential treatment effects [42, 43, 44].

## Limitations

This retrospective, single-center study has several limitations. Despite standardized local protocols, residual selection bias cannot be excluded, as IVT decisions remained at the discretion of treating physicians after the time-based switch from rt-PA to TNK. The small cohort size limits statistical power, particularly for rare outcomes such as sICH; with the observed event rate of ~ 2.5%, only large absolute differences (~ 20% points) would have been statistically detectable. Accordingly, the analyses are exploratory, and non-significant results should be interpreted as limited precision rather than proof of equivalence. Moreover, outcomes were assessed only during hospitalization without long-term functional follow-up, restricting evaluation of efficacy. Finally, the generalizability of our findings is limited by center-specific imaging pathways and DOAC assay protocols. Larger, prospective multicenter studies with standardized follow-up are warranted to confirm these observations.

## Conclusions

Our study indicates a similar safety profile of TNK based IVT in AIS-patients with DOAC compared to rt-PA. Since we overall found no evidence for an increased rate of IVT-associated complications, we recommend implementation of specific SOPs for IVT in this patient population.

## Supplementary Information

Below is the link to the electronic supplementary material.

Supplementary Material 1

## Data Availability

The data that support the findings of this study are not publicly available due to ethical restrictions but may be available from the corresponding author upon reasonable request and with appropriate permissions.

## Abbreviations

AIS: Acute ischemic stroke
cCT: Cranial computed tomography
DOAC: Direct oral anticoagulants
EVT: Endovascular treatment
ICH: Intracranial hemorrhage
INR: International normalized ratio
IQR: Interquartile range
IVT: Intravenous thrombolysis
LVO: Large vessel occlusion
mRS: Modified Rankin Scale
mTICI: Modified Thrombolysis in Cerebral Infarction
NIHSS: National Institutes of Health Stroke Scale
PH: Parenchymatous hematoma
rt-PA: Alteplase
SD: Standard deviation
sICH: Symptomatic intracranial hemorrhage
SOP: Standard operating procedure
TNK: Tenecteplase

## References

[1] Ringleb, P., Bauer, G., & Purrucker, J. (2023). Intravenöse thrombolyse des ischämischen Schlaganfalls – aktueller stand. *Der Nervenarzt*, *94*(6), 551–563. 37249597 10.1007/s00115-023-01500-9

[2] Meinel, T. R., et al. (2023). Intravenous thrombolysis in patients with ischemic stroke and recent ingestion of direct oral anticoagulants. *JAMA Neurol*, *80*(3), 233–243. 36807495 10.1001/jamaneurol.2022.4782 PMC9857462

[3] Seiffge, D. J., et al. (2021). Recanalisation therapies for acute ischaemic stroke in patients on direct oral anticoagulants. *Journal of Neurology, Neurosurgery and Psychiatry*, *92*(5), 534–541. 33542084 10.1136/jnnp-2020-325456 PMC8053326

[4] Kristoffersen, E. S., Seiffge, D. J., & Meinel, T. R. (2024). Intravenous thrombolysis and mechanical thrombectomy in acute stroke patients on direct oral anticoagulants. *Journal of Neurology*, *272*(1), 82. 39708167 10.1007/s00415-024-12832-0 PMC11663162

[5] Emberson, J., et al. (2014). Effect of treatment delay, age, and stroke severity on the effects of intravenous thrombolysis with Alteplase for acute ischaemic stroke: A meta-analysis of individual patient data from randomised trials. *Lancet*, *384*(9958), 1929–1935. 25106063 10.1016/S0140-6736(14)60584-5 PMC4441266

[6] Ranta, A. (2024). The time has come. The time is now. IV Alteplase, will you please go now? *Neurology*, *103*(9), e209961. 39413336 10.1212/WNL.0000000000209961

[7] Warach, S. J., et al. (2023). Symptomatic intracranial hemorrhage with tenecteplase vs Alteplase in patients with acute ischemic stroke: The comparative effectiveness of routine tenecteplase vs Alteplase in acute ischemic stroke (CERTAIN) collaboration. *JAMA Neurol*, *80*(7), 732–738. 37252708 10.1001/jamaneurol.2023.1449 PMC10230371

[8] Mahawish, K., et al. (2021). Switching to tenecteplase for stroke thrombolysis: Real-world experience and outcomes in a regional stroke network. *Stroke*, *52*(10), e590–e593. 34465202 10.1161/STROKEAHA.121.035931

[9] Wang, L., et al. (2024). Comprehensive review of tenecteplase for thrombolysis in acute ischemic stroke. *J Am Heart Assoc*, *13*(9), e031692. 38686848 10.1161/JAHA.123.031692 PMC11179942

[10] Menon, B. K., et al. (2022). Intravenous tenecteplase compared with Alteplase for acute ischaemic stroke in Canada (AcT): A pragmatic, multicentre, open-label, registry-linked, randomised, controlled, non-inferiority trial. *The Lancet*, *400*(10347), 161–169. 10.1016/S0140-6736(22)01054-6 35779553

[11] Muir, K. W., et al. (2024). Tenecteplase versus Alteplase for acute stroke within 4·5 h of onset (ATTEST-2): A randomised, parallel group, open-label trial. *Lancet Neurology*, *23*(11), 1087–1096. 39424558 10.1016/S1474-4422(24)00377-6

[12] Wang, Y., et al. (2023). Tenecteplase versus Alteplase in acute ischaemic cerebrovascular events (TRACE-2): A phase 3, multicentre, open-label, randomised controlled, non-inferiority trial. *Lancet*, *401*(10377), 645–654. 36774935 10.1016/S0140-6736(22)02600-9

[13] Zhong, C. S., et al. (2021). Routine use of tenecteplase for thrombolysis in acute ischemic stroke. *Stroke*, *52*(3), 1087–1090. 33588597 10.1161/STROKEAHA.120.030859

[14] Rose, D., et al. (2023). Complications of intravenous tenecteplase versus Alteplase for the treatment of acute ischemic stroke: A systematic review and meta-analysis. *Stroke*, *54*(5), 1192–1204. 36951049 10.1161/STROKEAHA.122.042335 PMC10133185

[15] Murphy, L. R., et al. (2023). Tenecteplase versus Alteplase for acute stroke: Mortality and bleeding complications. *Annals of Emergency Medicine*, *82*(6), 720–728. 37178103 10.1016/j.annemergmed.2023.03.022

[16] Miller, S. E., & Warach, S. J. (2023). Evolving thrombolytics: From Alteplase to tenecteplase. *Neurotherapeutics*, *20*(3), 664–678. 37273127 10.1007/s13311-023-01391-3 PMC10275840

[17] Alamowitch, S., et al. (2023). European stroke organisation (ESO) expedited recommendation on tenecteplase for acute ischaemic stroke. *Eur Stroke J*, *8*(1), 8–54. 37021186 10.1177/23969873221150022 PMC10069183

[18] Correa-Paz, C., et al. (2024). Pharmacological preclinical comparison of tenecteplase and Alteplase for the treatment of acute stroke. *Journal of Cerebral Blood Flow and Metabolism*, *44*(8), 1306–1318. 38436292 10.1177/0271678X241237427 PMC11342720

[19] Purrucker, J. C., et al. (2024). Thrombolysis for ischaemic stroke despite direct oral anticoagulation. *Stroke Vasc Neurol*. 10.1136/svn-2023-002727 PMC11732844 38296587

[20] Alam, K., et al. (2024). Assessing mortality and safety of IV thrombolysis in ischemic stroke patients on direct oral anticoagulants (DOACs): A systematic review and meta-analysis. *Clinical Neurology and Neurosurgery*, *246*, 108523. 39278007 10.1016/j.clineuro.2024.108523

[21] Berge, E., et al. (2021). European stroke organisation (ESO) guidelines on intravenous thrombolysis for acute ischaemic stroke. *Eur Stroke J*, *6*(1), I–lxii. 33817340 10.1177/2396987321989865 PMC7995316

[22] Powers, W. J., et al. (2019). Guidelines for the early management of patients with acute ischemic stroke: 2019 update to the 2018 guidelines for the early management of acute ischemic stroke: A guideline for healthcare professionals from the American heart association/American stroke association. *Stroke*, *50*(12), e344–e418. 31662037 10.1161/STR.0000000000000211

[23] Bücke, P. (2024). Intravenous thrombolysis in patients with recent intake of direct oral anticoagulants: A target trial analysis after the liberalization of institutional guidelines. *Eur Stroke J*, p. 23969873241252751. 10.1177/23969873241252751 PMC11569526 38738861

[24] Bergh, E., et al. (2022). Reasons and predictors of non-thrombolysis in patients with acute ischemic stroke admitted within 4.5 h. *Acta Neurologica Scandinavica*, *146*(1), 61–69. 35445395 10.1111/ane.13622 PMC9323435

[25] Tsai, T. Y., et al. (2024). Risk of bleeding following non-vitamin K antagonist oral anticoagulant use in patients with acute ischemic stroke treated with Alteplase. *JAMA Intern Med*, *184*(1), 37–45. 37983035 10.1001/jamainternmed.2023.6160 PMC10660269

[26] Ghannam, M., et al. (2023). Intravenous thrombolysis for acute ischemic stroke in patients with recent direct oral anticoagulant use: A systematic review and meta-analysis. *J Am Heart Assoc*, *12*(24), e031669. 38108256 10.1161/JAHA.123.031669 PMC10863770

[27] Kam, W., et al. (2022). Association of recent use of non–vitamin K antagonist oral anticoagulants with intracranial hemorrhage among patients with acute ischemic stroke treated with Alteplase. *Journal of the American Medical Association*, *327*(8), 760–771. 35143601 10.1001/jama.2022.0948 PMC8832308

[28] Kleeberg, A., et al. (2024). Hemorrhagic complications after stroke treatment with intravenous thrombolysis despite use of direct oral Anticoagulants: An observational study. *Therapeutic Advances in Neurological Disorders*, *17*, 17562864241276206. 39290529 10.1177/17562864241276206 PMC11406647

[29] Shahjouei, S., et al. (2020). Safety of intravenous thrombolysis among patients taking direct oral anticoagulants: A systematic review and meta-analysis. *Stroke*, *51*(2), 533–541. 31884908 10.1161/STROKEAHA.119.026426

[30] Monjazeb, S., Chang, H. V., & Lyden, P. D. (2024). Before, during, and after: An argument for safety and improved outcome of thrombolysis in acute ischemic stroke with direct oral anticoagulant treatment. *Annals of Neurology*, *96*(5), 871–886. 39258443 10.1002/ana.27058 PMC11496014

[31] Sekita, A., et al. (2025). Switch to tenecteplase for intravenous thrombolysis in stroke patients: Experience from a German high-volume stroke center. *Neurological Research and Practice*, *7*(1), 28. 40320554 10.1186/s42466-025-00388-x PMC12051303

[32] Wahlgren, N., et al. (2007). Thrombolysis with Alteplase for acute ischaemic stroke in the safe implementation of thrombolysis in stroke-monitoring study (SITS-MOST): An observational study. *Lancet*, *369*(9558), 275–282. 17258667 10.1016/S0140-6736(07)60149-4

[33] Schulman, S. and C. Kearon. (2005). Definition of major bleeding in clinical investigations of antihemostatic medicinal products in non‐surgical patients. *Journal of Thrombosis and Haemostasis*, *3*(4), 692–694. 10.1111/j.1538-7836.2005.01204.x 15842354

[34] Matusevicius, M. (2025). Intravenous Thrombolysis in Patients Taking Direct Oral Anticoagulation Treatment Before Stroke Onset: Results from the Safe Implementations of Treatments in Stroke International Stroke Registry. *Ann Neurol*. 10.1002/ana.27189 PMC12082013 39902556

[35] Behnoush, A. H., et al. (2023). Meta-analysis of outcomes following intravenous thrombolysis in patients with ischemic stroke on direct oral anticoagulants. *Bmc Neurology*, *23*(1), 440. 38102548 10.1186/s12883-023-03498-8 PMC10722877

[36] Koscumb, P., et al. (2024). Support for thrombolytic therapy for acute stroke patients on direct oral anticoagulants: Mortality and bleeding complications. *West J Emerg Med*, *25*(3), 399–406. 38801047 10.5811/westjem.18063 PMC11112660

[37] Katsanos, A. H., et al. (2021). Intravenous thrombolysis with tenecteplase in patients with large vessel occlusions: Systematic review and meta-analysis. *Stroke*, *52*(1), 308–312. 33272127 10.1161/STROKEAHA.120.030220

[38] Kheiri, B., et al. (2018). Tenecteplase versus Alteplase for management of acute ischemic stroke: A pairwise and network meta-analysis of randomized clinical trials. *Journal of Thrombosis and Thrombolysis*, *46*(4), 440–450. 30117036 10.1007/s11239-018-1721-3

[39] Amundsen, E. K., et al. (2024). Acute ischemic stroke and measurement of Apixaban and rivaroxaban: An observational cohort implementation study. *Res Pract Thromb Haemost*, *8*(1), 102307. 38314168 10.1016/j.rpth.2023.102307 PMC10837088

[40] Beyer-Westendorf, J., Fay, M., & Amara, W. (2021). The importance of appropriate dosing of nonvitamin K antagonist oral anticoagulants for stroke prevention in patients with atrial fibrillation. *TH Open*, *05*(03), e353–e362. 10.1055/s-0041-1731777 PMC8382498 34435170

[41] Xiong, Y., et al. (2024). Rationale and design of tenecteplase reperfusion therapy in acute ischaemic cerebrovascular events III (TRACE III): A randomised, phase III, open-label, controlled trial. *Stroke Vasc Neurol*, *9*(1), 82–89. 37247876 10.1136/svn-2023-002310 PMC10956103

[42] Yogendrakumar, V., et al. (2023). Tenecteplase improves reperfusion across time in large vessel stroke. *Annals of Neurology*, *93*(3), 489–499. 36394101 10.1002/ana.26547

[43] Badger, G., et al. (2025). Early recanalization and distal thrombus migration in large vessel occlusion stroke undergoing bridging thrombolysis with Alteplase versus tenecteplase (S22.010). *Neurology*, *104*(7_Supplement_1), 3502–p.

[44] Macha, K., et al. (2019). Cerebral ischemia in patients on direct oral anticoagulants. *Stroke*, *50*(4), 873–879. 30852963 10.1161/STROKEAHA.118.023877

