# Supplementary material for: Safety of tenecteplase versus alteplase for intravenous thrombolysis in acute ischemic stroke patients with direct oral anticoagulation: experience from a German stroke center
Source: Neurol Res Pract. 2025 Nov 14;7(1):88. doi: 10.1186/s42466-025-00450-8 (PMC12619176; doi:10.1186/s42466-025-00450-8)
Supplement: Supplementary file 1 — Supplementary Material 1 [file 42466_2025_450_MOESM1_ESM.docx]

**Supplemental Material**

Mers L. et al. *Safety of Tenecteplase Versus Alteplase for Intravenous Thrombolysis in Acute Ischemic Stroke Patients with Direct Oral Anticoagulation: Experience From a German Stroke Center*

**Content:**

**Supplemental Table 1:** Subgroup analyses according to time-window of IVT, DOAC-level and recanalization treatment

**Supplemental Table 2:** Propensity Score Matching Analyses

1. Post Propensity Score Matching Analysis for baseline characteristics
2. Post Propensity Score Matching Analysis for hemorrhagic complications and clinical outcomes

**Supplemental Figure 1:** In-hospital safety and clinical outcomes

1. Radiological and clinical outcomes comparing TNK and rt-PA treated patients.
2. Clinical course during hospitalization comparing TNK and rt-PA treated patients.

**Supplemental Figure 2:** Score distribution on the Modified Rankin Scale at discharge comparing TNK and rt-PA treated patients.

**Supplemental Table 1:**  **Subgroup analyses according to time-window of IVT, DOAC-level and recanalization treatment**

| **Subgroup** | **Any intracerebral hemorrhage**  **n = 6**  **(7.3 %)** | | **Early major NIHSS improvement**  **n = 35**  **(43.8 %)*** | |
| --- | --- | --- | --- | --- |
|  | **TNK**  **n = 4** | **rt-PA**  **n = 2** | **TNK**  **n = 17** | **rt-PA**  **n = 19** |
| **IVT-time window** |  |  |  |  |
| ≤ 4.5 h; n = 50 (61.0 %) | 2/28 (7.1 %) | 2/22 (9.1 %) | 14/28 (50.0 %) | 10/22 (45.5 %) |
| > 4.5 h; n = 32 (39.0 %) | 2/14 (14.3 %) | 0/18 | 3/14 (21.4 %) | 9/18 (50.0 %) |
| **Drug specific DOAC level**** |  |  |  |  |
| ≤ 50 ng/mL; n = 48 (59.3 %) | 1/22 (4.5 %) | 1/27 (3.7 %) | 10/22 (45.5 %) | 13/27 48.1 %) |
| > 50 ng/mL; n = 33 (40.7 %) | 3/20 (15.0 %) | 1/12 (8.3 %) | 7/20 (35.0 %) | 5/12 (41.7 %) |
| **Recanalization treatment** |  |  |  |  |
| IVT and EVT; n = 26 (31.7 %) | 0/13 | 0/13 | 7/13 (53.8 %) | 8/13 (61.5 %) |
| IVT only; n = 56 (68.3 %) | 4/29 (13.8 %) | 2/27 (7.4 %) | 10/29 (34.5 %) | 11/27 (40.7 %) |

* defined as NIHSS score improvement ≥4 points or NIHSS 0 within 72 h after admission.

** One patient from the rt-PA-group is excluded due to missing drug specific DOAC level, ng/mL.

Abbreviations: DOAC indicates direct oral anticoagulant; EVT, endovascular treatment; IVT, intravenous thrombolysis; rt-PA, alteplase; TNK, tenecteplase.

**Supplemental Table 2: Propensity Score Matching Analyses**

1. **Analyses for baseline characteristics**
2. **Analyses for hemorrhagic complications and clinical outcomes**

**Table 2a**

| **Parameter** | **AIS-patients on DOAC treated with IVT** | | ***p* Value** |
| --- | --- | --- | --- |
|  | **TNK**  **n = 30** | **rt-PA**  **n = 30** |  |
| Age, y; median (IQR) | 82 (71-85) | 81 (74-85) | 0.8 |
| Female sex; n (%) | 16 (53.3 %) | 14 (46.7 %) | 0.6 |
| **Prior medical history** |  |  |  |
| Previous Stroke or TIA; n (%) | 10 (33.3 %) | 9 (30.0 %) | 0.7 |
| Coronary heart disease; n (%) | 6 (20.0 %) | 8 (26.7 %) | 0.5 |
| Heart failure; n (%) | 4 (13.3 %) | 2 (6.7 %) | 0.4 |
| Hypertension; n (%) | 27 (90.0 %) | 27 (90.0 %) | 1.0 |
| Diabetes mellitus Type II; n (%) | 9 (30.0 %) | 10 (33.3 %) | 1.0 |
| Vascular disease*; n (%) | 5 (16.7 %) | 7 (23.3 %) | 0.7 |
| Atrial fibrillation; n (%) | 27 (90 %) | 27 (90 %) | 1.0 |
| **DOAC characteristics** |  |  |  |
| Agent; n (%) |  |  |  |
| - Apixaban | 16 (53.3 %) | 19 (63.3 %) | 0.4 |
| - Edoxaban | 10 (33.3 %) | 6 (20.0 %) | 0.2 |
| - Rivaroxaban | 4 (13.3 %) | 3 (10.0 %) | 1.0 |
| - Dabigatran | 0 | 2 (6.7 %) | 0.5 |
| Drug specific DOAC level, ng/mL; median (IQR) | 38 (24-79) | 30 (22-71) | 0.4 |
| **Stroke characteristics** |  |  |  |
| NIHSS on admission; median (IQR) | 9 (4-14) | 8 (4-13) | 0.6 |
| Large vessel occlusion**; n (%) | 16 (53.3 %) | 16 (53.3 %) | 1.0 |

*defined as the presence of coronary artery disease, peripheral artery disease or aortic plaque

** Internal Carotid Artery, M1/M2 segment of the Middle Cerebral Artery, Posterior Cerebral Artery, Vertebral Artery, Basilar Artery

Abbreviations: AIS indicates acute ischemic stroke; DOAC, direct oral anticoagulant; IQR, interquartile range; IVT, intravenous thrombolysis; mRS, modified Rankin Scale; NIHSS, National Institutes of Health Stroke Scale; TIA, transient ischemic attack; rt-PA, alteplase; TNK, tenecteplase

**Table 2b**

| **Parameter** | **AIS-patients on DOAC treated with IVT** | | ***p* Value** |
| --- | --- | --- | --- |
|  | **TNK**  **n = 30** | **rt-PA**  **n = 30** |  |
| **Hemorrhagic complications; n (%)** |  |  |  |
| Any intracerebral hemorrhage | 2 (6.7 %) | 2 (6.7 %) | 1.0 |
| Symptomatic intracerebral hemorrhage (SITS-MOST criteria) | 0 | 1 (3.3 %) | 1.0 |
| **Any complication; n (%)** | 2 (6.7 %) | 3 (10.0 %) | 1.0 |
| **Clinical outcomes; n (%)** |  |  |  |
| Early major NIHSS improvement (≥ 4 points within 72 h or NIHSS = 0) | 13 (43.3 %) | 14 (46.7 %) | 0.8 |
| Early major NIHSS deterioration (≥ 4 points within 72 h) | 2 (6.7 %) | 4 (13.3 %) | 0.7 |

Abbreviations: AIS indicates acute ischemic stroke; DOAC, direct oral anticoagulant; IVT, intravenous thrombolysis; NIHSS, National Institutes of Health Stroke Scale; rt-PA, alteplase; TNK, tenecteplase.

**Supplemental Figure 1: In-hospital safety and clinical outcomes**

1. **Radiological and clinical outcomes comparing TNK and rt-PA treated patients.**
2. **Clinical course during hospitalization comparing TNK and rt-PA treated patients.**


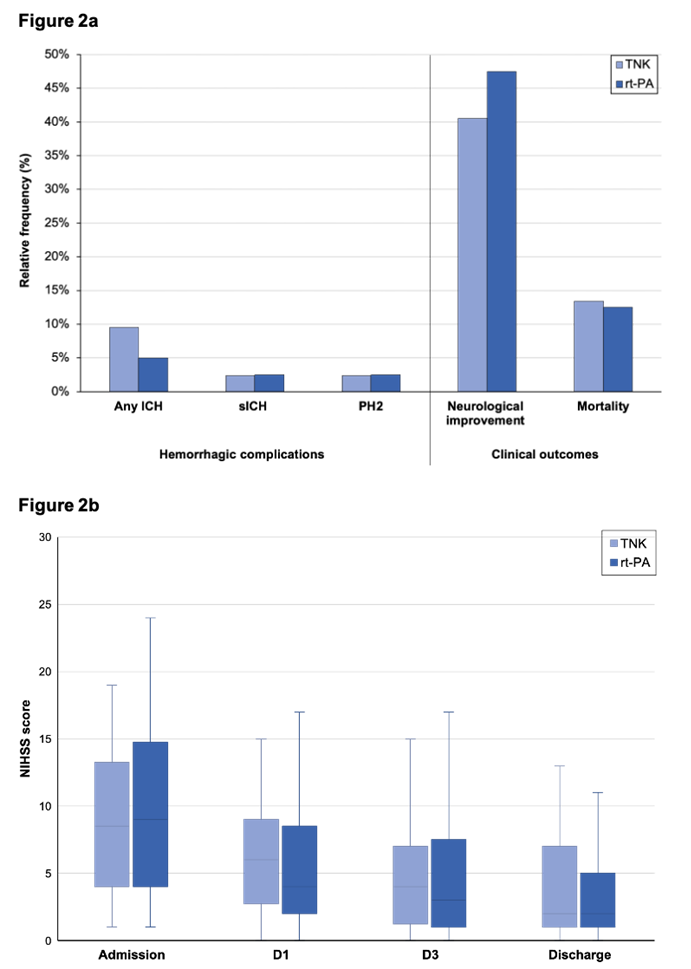


Figure 2a) Hemorrhagic complications were categorized into any ICH, symptomatic ICH according to SITS-MOST criteria, and parenchymatous hematoma according to the Heidelberg Bleeding Classification. Clinical outcomes are categorized into neurological improvement (i.e., NIHSS improvement ≥ 4 points or NIHSS 0 within 72 h after admission) and mortality. Figure 2b) Boxplot represents the distribution of NIHSS score differences between both groups according to fibrinolytic agent after 24 h, 72 h and at discharge compared with baseline. Blue boxes indicate the interquartile range including median. Minimum and maximum are represented by whiskers. Post-EVT intubated patients and deceased patients were excluded. Abbreviations: ICH indicates intracranial hemorrhage; PH, parenchymatous hematoma; rt-PA, alteplase; sICH, symptomatic intracranial hemorrhage; TNK, tenecteplase, NIHSS, National Institutes of Health Stroke Scale; D, day.

**Supplemental Figure 2: Score distribution on the Modified Rankin Scale at discharge comparing TNK and rt-PA treated patients.**


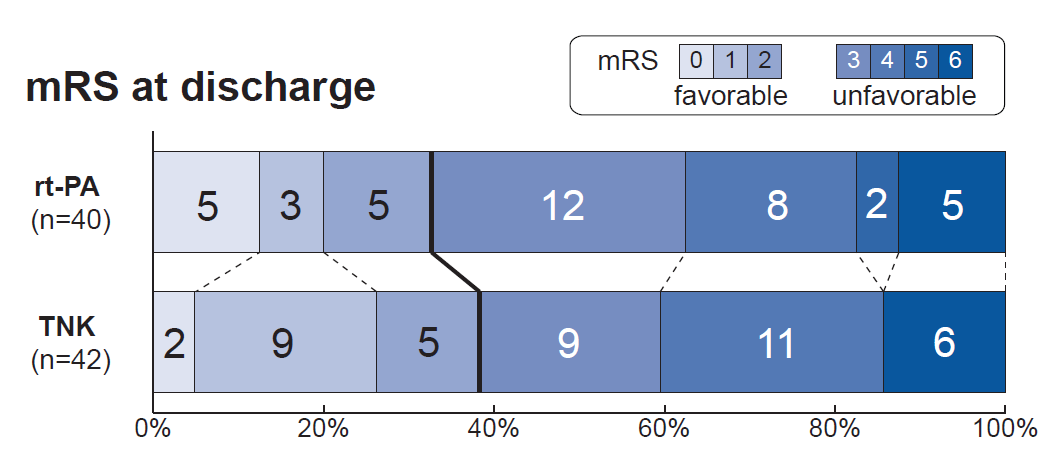


Absolute frequencies are shown as numbers, and bar sections indicate the relative distribution of modified Rankin Scale (mRS) scores at discharge in patients receiving rt-PA versus TNK. The score distribution is dichotomized into favorable (i.e., mRS = 0-2) and unfavorable (i.e., mRS = 3-6) as indicated by the thick line. Abbreviations: mRS indicates modified Rankin Scale; rt-PA, alteplase; TNK, tenecteplase.
